# Dysfunctional Attitudes Mediate the Relationship Between Childhood Emotional Neglect and Anhedonia in Young Adult Major Depression Patients

**DOI:** 10.3389/fpsyt.2022.791230

**Published:** 2022-01-27

**Authors:** Peilin Wang, Nan Zhang, Simeng Ma, Lijun Kang, Wei Wang, Xiaofen Zong, Hanping Bai, Ruiting Li, Zhongchun Liu

**Affiliations:** Department of Psychiatry, Renmin Hospital of Wuhan University, Wuhan, China

**Keywords:** depression, anhedonia, dysfunctional attitudes, structural equation model, childhood trauma

## Abstract

**Background:**

Childhood traumas are well-established risk factors for major depressive disorder (MDD). However, the relationship between childhood traumas types and MDD symptoms is unclear. The present study tested the hypothesis that childhood traumas affect specific types of anhedonia in depression and the mediating role of dysfunctional attitude.

**Methods:**

Within this cross-sectional study, 310 young adult patients with MDD completed the PHQ-9, CTQ-SF, DAS, and SHAPS. The statistical analyses used the Mann-Whitney U test, Spearman's rank correlation, and multiple regression analysis. Mediation analyses were tested by the structural equation model (SEM).

**Results:**

Spearman's rank correlation analysis showed positive correlations between the SHAPS, CTQ-SF, and DAS total score (*p* < 0.05). The EA, EN, PN, and SHAPS scores were positively correlated (*p* < 0.05). Among the four factors of anhedonia, social interaction and interest/pastimes were positively correlated with EA, EN, and PN (*p* < 0.05), the sensory experience was positively correlated with EN (*p* < 0.01), and diet did not correlate with childhood traumas. Stepwise regression analysis showed that dysfunctional attitude and emotional neglect were the main influencing factors of sensory experience (*p* < 0.001) and social interaction (*p* < 0.001). Dysfunctional attitude and physical neglect were the main factors influencing interest/pastimes (*p* < 0.001). SEM analysis found that dysfunctional mediated between childhood traumas and anhedonia.

**Conclusions:**

The degree of anhedonia was related to dysfunctional attitudes and childhood traumas. The childhood emotional neglect experience was the most important and was related to sensory and social anhedonia. Dysfunctional attitudes played a mediating role between childhood neglect and anhedonia. Early psychotherapy targeting young adult MDD patients with childhood emotional neglect may help decrease symptoms of anhedonia.

## Introduction

Anhedonia is one of the core features of major depressive disorder (MDD), according to the Diagnostic and Statistical Manual of Mental Disorders (DSM-5) (1). Anhedonia refers to people's loss of the ability to experience happiness or a decline in the ability to experience happiness, including lack of pleasure in interest and success (2). The presence of symptoms of anhedonia, as a predictor of poor treatment response (3), can change independently of other symptoms of depression (4, 5).

Childhood traumas have become major social public-health problems worldwide (6, 7). As critical early-life adverse events, childhood traumas link to various adult psychopathologies, such as the first onset of depression (8, 9), bipolar disorder, anxiety, psychosis, disruptive behavior, substance abuse, and eating disorders (10, 11). Further, childhood maltreatment exposures predict a more chronic, treatment-resistant, and severe depression than individuals with depression but without a maltreatment history (12). Among Chinese young adults, the prevalence rate of childhood traumas exposures is as high as 18.6% (13). Several studies revealed the association between childhood traumas and high anhedonia (14, 15). These studies suggested that when considering the symptom dimension, childhood traumas may mainly affect the anhedonia aspect of MDD. Childhood traumas include physical abuse, emotional abuse, sexual abuse, physical neglect, and emotional neglect (13). At present, physical and emotional neglect has attracted more and more attention (7). Current knowledge about the impact of childhood traumas on specific anhedonia is limited, such as the state anhedonia (16) and social anhedonia (17).

According to Beck's schema-based cognitive model, an individual's emotional experiences depend on the content of thoughts and beliefs activated by life experiences (18). According to the diathesis-stress model, childhood traumas may cause vulnerable individuals to form negative cognitive schemas (19). These negative thinking styles are typically conceptualized as dysfunctional attitudes, rigid and maladaptive beliefs about oneself, the world, and the future (20, 21). Dysfunctional attitudes, as cognitive vulnerabilities, interact with adverse life events to affect depression (22). Current research showed that dysfunctional attitudes are not only related to the severity of depression and the risk of recurrence (23) but can also persist as stable features (24) and mediate the impact of childhood traumas on depression (25).

In the current study, we first determined the effects of different types of childhood traumas on different domains of anhedonia. Then examined the mediating role of dysfunctional attitudes between different types of childhood traumas and anhedonia. Our hypotheses were as follows: (a) specific types of childhood traumas were associated with severe anhedonia; (b) dysfunctional attitudes mediated the relationship between childhood traumas and anhedonia in MDD patients.

## Method

### Study Design and Participants

This study was based on China's Early Warning System and Comprehensive Intervention for Depression (ESCID) project from April 2019 to January 2020. A total of 310 participants were included. Two experienced psychiatrists diagnosed all participants and met the DSM-5 diagnostic criteria for major depressive disorder. The inclusion criteria of participants were: 18–30 years of age, having a junior high school education or higher. The exclusion criteria were as follows: other mental illnesses, substance dependence or abuse, severe physical illness or craniocerebral trauma, or severe excitement, impulsivity, or non-cooperation. This experiment was examined and approved by the Ethics Committee of the Renmin Hospital of Wuhan University. All the participants were informed and agreed to participate in this study.

### Measures

The nine-item Patient Health Questionnaire (PHQ-9) is a self-assessment questionnaire for patients with depression. The PHQ-9 has been widely used and is a valid measure of depression in clinical populations (26–28). The total score ranges from 0–27, with the following grades: no depression (0–4), mild depression (5–9), moderate depression (12–16), and severe depression (≥ 15) (29). In our study, the PHQ-9 demonstrated strong internal consistency (α = 0.875).

The Snaith-Hamilton Pleasure Scale (SHAPS) is a self-report scale containing 14 items designed to evaluate a person's enjoyment experience (food/drink, interest/pastimes, social interactions, and pleasurable sensory experiences) in the past few days (30). The SHAPS is not influenced by participants' demographic and clinical characteristics, possesses excellent psychometric properties, and appears appropriate for clinical and research settings (31). The Chinese version of SHAPS is answered according to a Likert-style 4-point system (1 point, absolutely agree; 2 points, agree; 3 points, disagree; 4 points, absolutely disagree), with a total score of 14 to 56 points (32). The higher the score, the more obvious anhedonia. According to the research of Zhang et al. (33), the Spanish four-factor structure (34) was used in this study: sensory experience (items 6, 7, 11, 12, 13), food/drink (items 4, 5, 9, 10), social interaction (items 2, 8, 14), and interest/pastimes (items 1, 3). This model provides the best fit for both Chinese non-clinical and clinical samples. In our study, Cronbach's α coefficients (α = 0.913) for the total SHAPS reached accepted standards (α > 0.70), and all four subscales scored above 0.60.

The Childhood Trauma Questionnaire-Short Form (CTQ-SF) is a self-report scale containing 28 items (25 clinical items and 3 validation items). It is used to retrospective evaluate traumatic experiences in childhood (35), including physical abuse (PA), emotional abuse (EA), sexual abuse (SA), physical neglect (PN), and emotional neglect (EN). It is a 5-point Likert scale, from “Never” (score = 1) to “Always” (score = 5). The total score ranges from 25 to 125 points. The higher the score, the higher the experience of abuse/neglect. According to previous studies (36), cutoff points for CTQ-SF subscales are EA score ≥ 13, PA score ≥ 10, SA score ≥ 8, EN score ≥ 15, and PN score ≥ 10, and CTQ-SF total ≥ 50. The Chinese version of CTQ-SF has good reliability and validity in college students and depression (37). In our study, Cronbach's α coefficients for the total CTQ-SF was 0.870, and all five subscales scored above 0.60, indicating that CTQ-SF has good structural validity in the Chinese depression sample.

The Chinese version of the Dysfunctional Attitude Scale (DAS) is a self-report scale consisting of 40 items. Designed to assess the cognitive vulnerability of depression, it may reflect the impact of early adverse events on a person's perception of self and the world. Each item includes a statement on the subject and a 7-point Likert scale to assess the degree of agreement (1 point = completely disagree; 7 points = completely agree). Among them, 10 items are scored in reverse (items 2, 6, 12, 18, 24, 29, 30, 35, 37, 40). The scoring range is 40 to 280 points, and the total normal score is ≤ 130 points. The higher the score, the more distorted the subject's cognition (38). DAS has good reliability and validity in Chinese MDD patients (13). In our study, Cronbach's α coefficients (α = 0.917) for the total DAS reached accepted standards (α > 0.70).

Suicidality was assessed by asking participants if they ever had suicidal ideation, plans, or attempts in their lifetime. Self-injury was assessed by asking participants if they ever had self-injury attempts in their lifetime. We asked about the use of the substance, including having ever used a substance, tobacco, or alcohol. Family history was assessed by asking participants whether their biological parents and siblings ever had depression.

### Statistical Analysis

Categorical data were expressed in frequency and percentage (N, %), and the Chi-square test was used to compare differences between groups. Kolmogorov-Smirnov test was used to test the normality of the continuous data (39), showing that the survey result data were non-normally distributed (*p* < 0.05). So non-normal data was represented by Median and IQR. The non-parametric Mann-Whitney *U*-test was used to compare differences between groups. Spearman's rank correlation test was used to explore the relationships between variables. Stepwise multiple linear regression analysis was used to test the impact of different types of childhood traumas and dysfunctional attitudes on anhedonia when demographic characteristics were controlled.

A structural equation model (SEM) including full information maximum likelihood (FIML) estimation was used to examine the mediation model with gender and age as covariates. Standardized direct, indirect, and total effects were estimated for all pathways. We calculated a 95% bootstrap confidence interval (*CI*) with 5000 bootstrapped samples to examine the significance of direct and indirect effects. The following fit criteria were used to evaluate the goodness-of-fit of the model (40): χ^2^*/df* ≤ 3, the root-mean-square error of approximation (RMSEA) ≤ 0.08, the Standardized Root-Mean-Square Residual (SRMR) ≤ 0.08 (41), the Comparative Fit Index (CFI) ≥ 0.90(42), the goodness of fit index (GFI) ≥ 0.90.

SPSS 25.0 was used for single factor and multivariate statistical analysis, and AMOS 23.0 was used for SEM analysis. A two-tailed significance level of overall *p* < 0.05 was considered statistically significant in this study.

## Results

### Participants' Socio-Demographic Characteristics

In total, 310 patients were participated, including 65 males (20.97%) and 245 females (79.03%). When a subscale score was higher than the cut-off point, we analyzed the frequencies of childhood traumas. In survey 175 (56.5%) participants reported at least one type of trauma. The prevalence rates of childhood emotional abuse (EA), physical abuse (PA), sexual abuse (SA), emotional neglect (EN), and physical neglect (PN) were 24.52, 17.1, 11.94, and 41.61, and 31.61%. Among those people, 85 (37.1%) participants reported more than one type of trauma. The two most reported trauma types were emotional neglect (*n* = 129, 41.61%) and physical neglect (*n* = 98, 31.61%).

According to CTQ-SF total ≥ 50, patients were divided into no childhood trauma group (*n* = 100, 32.3%) and childhood traumas group (*n* = 210, 67.7%), and the socio-demographic differences between the two groups were compared (Table 1). Patients with childhood traumas showed more childhood separation from their parents (*p* < 0.001), a lower education level (*p* = 0.026), higher substance use (*p* < 0.001), more suicide plans/behaviors in the lifetime (*p* < 0.001), and more self-harm behaviors (*p* = 0.023). Although there was no significant difference in age between the two groups (*p* = 0.188), patients with childhood traumas had younger onset age (*p* = 0.001) with a longer duration of illness (*p* = 0.003). It showed that childhood traumas were associated with early-onset depression. We also assessed the gender differences in five childhood traumas (Table 2) and found that the prevalence of EA in female patients was higher than males (χ^2^ =10.384, *p* = 0.001^**^). Therefore, age, gender, and education level were used as control variables in the subsequent analysis.

**Table 1:** The socio-demographic and clinical characteristics.

	**No CT (***n =*** 210)**	**CT (***n =*** 100)**		
**Variables**	**N (%)**	**N (%)**	***χ^2^***	***p***
Female	159 (64.90%)	86 (35.10%)	4.325	0.038*
First episode	156 (67.20%)	76 (32.80%)	0.106	0.745
**Education level**				
Undergraduate	176 (65.40%)	93 (34.60%)	4.986	0.026*
Graduate	34 (82.90%)	7 (17.10%)		
**Residence**				
City	164 (68.30%)	76 (31.70%)	0.234	0.890
Town	32 (66.70%)	16 (33.30%)		
Village	14 (63.60%)	8 (36.40%)		
Separated from parents	45 (48.90%)	47 (51.10%)	21.225	<0.001***
Substance use in the lifetime	12 (34.30%)	23 (65.70%)	20.058	<0.001***
Suicide^a^	58 (54.20%)	49 (45.80%)	13.701	<0.001***
Self-injury^b^	59 (59.00%)	41 (41.00%)	5.163	0.023*
Family history	28 (60.90%)	18 (39.10%)	1.167	0.280
**Variables**	**Median (IQR)**	**Median (IQR)**	***Mann-Whitney U***	***p***
Age (year)	22 (21,23)	21 (20,23)	9,511.5	0.188
Age of onset (year)	19 (17,20)	17 (15,19)	8,054	0.001**
Duration of illness (year)	1 (0,2)	2 (1,3)	8,310	0.003**
HAMD-17	19 (12,23)	21 (16,25)	8,151	0.001**
PHQ-9	16 (10,20)	18 (14,23)	8,301.5	0.003**
DAS	163 (146,182)	176 (159,197)	7,736.5	<0.001***
SHAPS	31 (27,36)	35 (28,39)	8,280.5	0.003**
Sensory experience	11 (10,13)	12 (10,14)	8,530	0.007**
Food/drink	9 (8,11)	10 (8,11)	9,479.5	0.162
Social interaction	6 (5,7)	7 (6,8)	7,730.5	<0.001***
Interest/pastimes	4 (4,5)	5 (4,6)	8,743	0.014*
CTQ-SF scores	36 (31,41)	61 (54,68)	0	<0.001***
EA	7 (6,9)	14 (11,18)	1,859	<0.001***
PA	5 (5,7)	8 (6,13)	4,442.5	<0.001***
SA	5 (5,5)	5 (5,8)	7,351.5	<0.001***
EN	10 (7,13)	20 (17,21)	785.5	<0.001***
PN	6 (5,8)	11 (9,14)	2,239	<0.001***

* *p < 0.05*,

** *p < 0.01*,

*** *p < 0.001 (2-tailed)*.

a *Suicide plans/behaviors in the life time*.

b *Self-injury behaviors in the life time. PHQ-9, nine-item Patient Health Questionnaire; SHAPS, Snaith-Hamilton Pleasure Scale; CTQ-SF, Childhood Trauma Questionnaire–Short Form; EA, emotional abuse; PA, physical abuse; SA, sexual abuse; EN, emotional neglect; PN, physical neglect; DAS, Dysfunctional attitude scale*.

**Table 2:** Prevalence of five childhood traumas in different genders.

	**Female (*n =* 245)**	**Male (*n =* 65)**	***χ^2^***	***p***
EA	70 (28.6%)	6 (9.2%)	10.384	0.001**
PA	46 (18.8%)	7 (10.8%)	2.323	0.127
SA	33 (13.5%)	4 (6.2%)	2.616	0.106
EN	105 (42.9%)	24 (36.9%)	0.745	0.388
PN	77 (31.4%)	21 (32.3%)	0.018	0.892

** *p < 0.01 (2-tailed). EA, emotional abuse; PA, physical abuse; SA, sexual abuse; EN, emotional neglect; PN, physical neglect*.

### Correlation's Analysis

Spearman's rank correlation tests were used to analyze the relationship between childhood traumas, anhedonia, and dysfunctional attitudes with age, gender, and education level as control variables. There were positive correlations between SHAPS total score, DAS total score, and CTQ-SF total score (*rho* = 0.150–0.254, *p* < 0.001–0.01). SHAPS total score was positively correlated with EA, EN, and PN scores (*rho* = 0.113–0.186, *p* < 0.01–0.05). Therefore, these three items were included in the subsequent analysis. Among the four factors of anhedonia, social interaction and interest/pastimes were positively correlated with EA, EN, and PN (*rho* = 0.121–0.223, *p* < 0.001–0.05), the sensory experience was positively correlated with EN (*rho* = 0.177, *p* < 0.01), however, food/drink was not correlated with childhood traumas. DAS total score was positively correlated with 4 factors of SHAPS (*rho* = 0.174–0.262, *p* < 0.001–0.01), and CTQ-SF total score and 4 factors (*rho* = 0.159–0.239, *p* < 0.001–0.01) except SA (*rho* = 0.106, *p* = 0.063). Complete results of the correlation analysis between childhood traumas and other scales are shown in Table 3.

**Table 3:** Spearman's rank correlation between childhood traumas, anhedonia and dysfunctional attitudes.

	**SHAPS**	**Sensory experience**	**Food/drink**	**Social interaction**	**Interest/pastimes**	**DAS**
EA	0.113*	0.084	0.054	0.163**	0.121*	0.238***
PA	0.076	0.062	0.015	0.122*	0.083	0.211***
SA	−0.045	−0.055	−0.025	−0.069	0.012	0.106
EN	0.186**	0.177**	0.112	0.223***	0.137*	0.159**
PN	0.141*	0.112	0.079	0.142*	0.202***	0.163**
CTQ-SF	0.15**	0.126*	0.077	0.187**	0.158**	0.239***
DAS	0.254***	0.246***	0.262***	0.174**	0.18**	1

* *p < 0.05*,

** *p < 0.01*,

*** *p < 0.001 (2-tailed). Age, gender, and education level as control variables. SHAPS, Snaith-Hamilton Pleasure Scale; CTQ-SF, Childhood Trauma Questionnaire–Short Form; EA, emotional abuse; PA, physical abuse; SA, sexual abuse; EN, emotional neglect; PN, physical neglect; DAS, Dysfunctional attitude scale*.

### Multiple Regressions Analysis

Four stepwise multiple linear regression models were calculated with the SHAPS total score and four subscales: sensory experience, social interaction, and interest/pastimes as the dependent variable. The independent variables included the DAS total score, EA, EN, PN, age, gender, and education level. The results showed that dysfunctional attitudes and emotional neglect were the main influencing factors of SHAPS total score (*R*^2^ = 0.092^***^), sensory experience (*R*^2^ = 0.084^***^), and social interaction (*R*^2^ = 0.092^***^). Dysfunctional attitudes and physical neglect were the main influencing factors of interest/pastimes (*R*^2^ = 0.065^***^). Table 4 shows the complete results of the multiple regressions.

**Table 4:** Stepwise multiple linear regressions analyses: associations of childhood traumas and dysfunctional attitudes with anhedonia.

	**Predictors**	**Unstandardized coefficients**		**Standardized coefficients**		
**Dependent**	**Independent**	**β**	***SE***	***B(95%CI)***	***t***	***R^**2**^***
SHAPS	DAS	0.061	0.014	0.240(0.033–0.089)	4.343***	0.092***
	EN	0.194	0.071	0.150(0.054–0.335)	2.718**	
Sensory experience	DAS	0.023	0.006	0.230(0.012–0.034)	4.151***	0.084***
	EN	0.073	0.029	0.142(0.017–0.130)	2.562*	
Social interaction	EN	0.065	0.018	0.197(0.029–0.101)	3.564***	0.092***
	DAS	0.009	0.004	0.134(0.002–0.016)	2.399*	
Interest/pastimes	PN	0.068	0.022	0.173(0.025–0.112)	3.09**	0.065***
	DAS	0.008	0.003	0.162(0.002–0.013)	2.901**	

* *p < 0.05*,

** *p < 0.01*,

*** *p < 0.001 (2-tailed). SHAPS, Snaith-Hamilton Pleasure Scale; EA, emotional abuse; PA, physical abuse; SA, sexual abuse; EN, emotional neglect; PN, physical neglect; DAS, Dysfunctional attitude scale*.

### Mediating Effect Analysis

To test the mediating role of dysfunctional attitudes between childhood traumas and anhedonia, we used SEM to test two mediation models with DAS being the mediator. We first included the overall sample, and then we separately analyzed in female and male groups. Age was controlled in all models.

Model 1 examined the mediating role of dysfunctional attitudes between EN and anhedonia. Since food/drink and childhood traumas were not significantly correlated, and EN was not included in the regression equation of interest/pastimes, only sensory experience and social interaction were taken as latent variable models of anhedonia.

**Figure 1:**
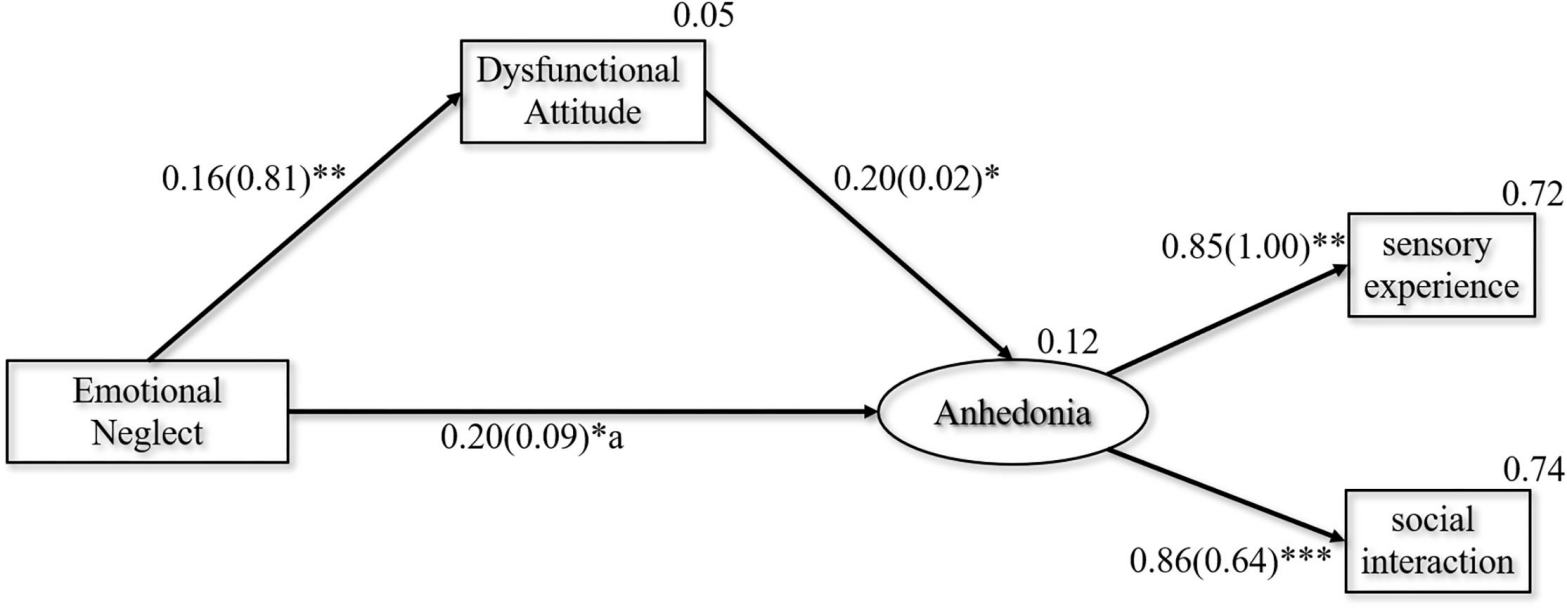
Final path model with the standardized and unstandardized coefficients presented in the parentheses. χ^2^*/df* = 2.331, GFI = 0.991, CFI = 0.986, RMSEA = 0.066, SRMR = 0.0232. *p < 0.05, **p < 0.01, ***p < 0.001.

As shown in Figure 1, the overall model showed a good fit: χ^2^*/df* = 2.331, GFI = 0.991, CFI = 0.986, RMSEA = 0.066, SRMR = 0.0232. The SEM analysis revealed that a standardized total effect of EN on anhedonia was 0.231 (*95% CI* (0.075, 0.357), *p* = 0.005), with the significant direct effect of EN on anhedonia being 0.198 (*95% CI* (0.034, 0.329), *p* = 0.018), and the significant indirect effect being 0.033 (*95% CI* (0.004, 0.081), *p* = 0.020) in the pathway of EN-DAS-anhedonia. This indirect effect suggested that higher EN increased the anhedonia via the DAS. In addition, we found that dysfunctional attitudes also had a partial mediation role in female patients (see Supplementary Figure 1). However, there was no significant correlation between EN, DAS, and anhedonia in male patients (see Supplementary Figure 2).

**Figure 2:**
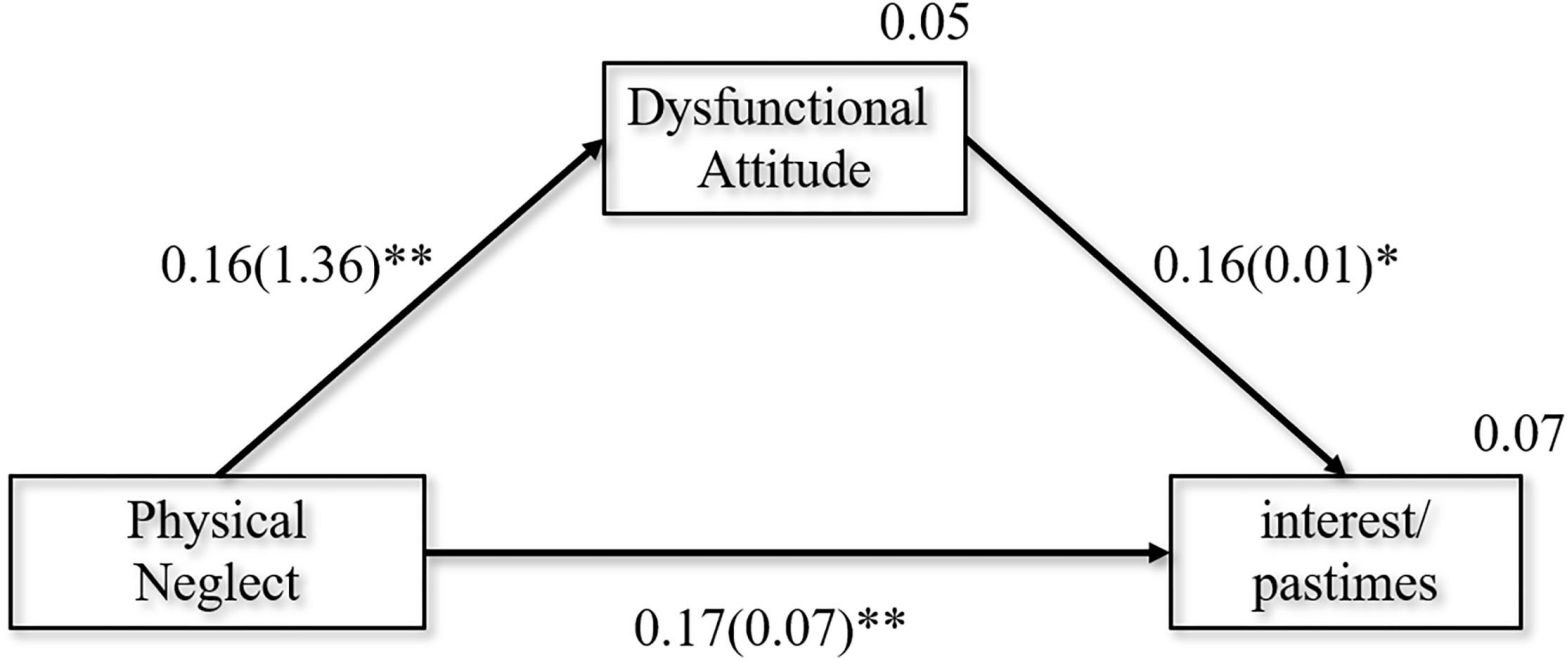
Final path model with the standardized and unstandardized coefficients presented in the parentheses. χ^2^*/df* = 0.409, GFI = 0.999, CFI = 1.000, SRMR = 0.0120. *p < 0.05, **p < 0.01.

Model 2 examined the mediating role of dysfunctional attitudes between PN and interest/pastimes. As shown in Figure 2, the overall model showed a good fit: χ^2^*/df* = 0.409, GFI = 0.999, CFI = 1.000, SRMR = 0.0120. The SEM analysis revealed that a standardized total effect of PN on interest/pastimes was 0.198 (*95% CI* (0.088, 0.303), *p* =0.001), with the significant direct effect of PN on interest/pastimes being 0.173 (*95% CI* (0.065, 0.282), *p* = 0.002), and the significant indirect effect being 0.026 (*95% CI* (0.005, 0.062), *p* = 0.011) in the pathway of PN-DAS-interest/pastimes. This indirect effect suggested that higher PN increased the interest/pastimes via the DAS. In addition, dysfunctional attitudes also had a partial mediation role in female patients (see Supplementary Figure 3). Nevertheless, there was no significant correlation between PN, DAS, and anhedonia in male patients (see Supplementary Figure 4).

## Discussion

In the present study, we investigated if different types of childhood traumas were associated with different dimensions of anhedonia in depressive patients and the role of dysfunctional attitudes in contributing to childhood traumas and anhedonic symptoms. Our findings indicated that dysfunctional attitudes were significant mediators between childhood traumas and anhedonia, indicating cognitive dissonance as a mechanism of anhedonia caused by childhood traumas.

Our findings indicated that anhedonia was related to specific types of childhood traumas. The stronger association came from specific trauma types of emotional abuse and neglect and physical neglect. Among them, the incidence of childhood emotional neglect was the highest in both men (36.9%) and women (42.9%), with no gender difference. Emotional neglect also had a stronger correlation with anhedonia in depression (*rho* = 0.186, *p* < 0.01), consistent with the previous reports (43, 44). Emotional neglect often coexists with emotional abuse, collectively called Childhood Emotional Abuse and Neglect (CEAN). This kind of childhood emotional maltreatment, different from sexual or physical abuse, is more insidious but more common in depression patients (45, 46).

Interestingly, our study found that physical neglect was associated with interest/pastimes of anhedonia. At present, there are few studies on childhood physical neglect. Physical neglect is not associated with mental health problems in early adulthood, such as depression, anxiety, and stress (47). Therefore, the specific reasons for this association need to be further studied. These results reiterate the importance of distinguishing between childhood misfortunes and clusters of symptoms when describing the relationship between maltreatment and depression.

We found that childhood traumas had main effects on specific domains of consummatory anhedonia. Consummatory anhedonia includes the source of enjoying many things and being able to appreciate the positive stimuli entirely (48). Specifically, we found that individuals with childhood traumas had higher sensory experience, social interaction, and interest/pastimes but not food/drink than participants with a low level of childhood traumas. According to the contents engender pleasure, anhedonia includes physical and social anhedonia. Our analysis showed that childhood emotional neglect was related to sensory experience and social interaction. Sensory experience and social interaction reflect social anhedonia to some extent (49). Therefore, childhood traumas are more likely to cause social anhedonia in patients with depression. Patients with social anhedonia showed decreased social connections, decreased social functioning, and decreased returns from social interactions. Social anhedonia may play an etiological role in developing adolescent depression (50). It is associated with increased severity of depression and poor treatment response (51).

Our study suggested that dysfunctional attitudes mediated the impact of childhood traumas on anhedonia. A recent systematic review also showed that negative cognitive styles could be used as mediating factors in the association between children's emotional abuse and depression (52). The anhedonia-centered model of depressive vulnerability suggests that childhood decreases positive reinforcements and increases negative reinforcements lead to an individual's personality and cognition disorder (53). In comparison, Beck's theory focuses on negative schema and dysfunctional attitudes (54). Childhood maltreatment is deemed to act as a severe environmental risk that may contribute individuals to the development of cognitive vulnerabilities (55) through ruminating and negative reasoning. These abnormal cognitive schemata will become a risk factor for depressive symptoms in adolescence. In addition, other forms of cognitive patterns may also relate to the association between childhood adversities and affective symptoms. Mansueto et al. (56) found that childhood abuse or neglect may be related to negative metacognitive beliefs, mediating the association between childhood adversities and negative emotions. A systematic review suggested that repetitive negative style may be involved in the association between childhood traumas and psychological symptoms in a clinical and non-clinical population: childhood abuse is related to worry and rumination; in contrast, childhood neglect is related to rumination (57).

Therefore, correcting cognitive disorders can reduce the severity of anhedonia for young adult patients who report childhood adversity. Psychotherapy can help improve patients' functional attitude disorders, such as interpersonal psychotherapy (IPT) (58) and cognitive-behavioral therapy (CBT) (59). Considering that depression patients with childhood traumas may have a greater risk of recurrence or treatment resistance, clinicians should provide them with tailor-made interventions to reduce the severity of depressive symptoms. Sequential combination of psychotherapy has a relative advantage in preventing relapse/recurrence of depression (60).

In addition, we found the mediating role of dysfunctional attitudes in the overall sample and the female sample, but not in males since gender differences play an important role in childhood traumas and dysfunctional attitudes. Sonmez et al. (15) found that female adolescents with MDD have a more significant association between anhedonia and sexual abuse than males. Similarly, females with MDD have higher DAS scores and more severe cognitive distortion in seeking applause, dependence, and self-determination than males (61), suggesting that gender differences should be considered when providing interventions. Future research should analyze different gender groups separately.

Our results have some shortcomings. First, our study was a cross-sectional study with no further follow-up to assess patient outcomes, and the CTQ-SF is a recall questionnaire. There might be recall bias in our study's assessment of childhood traumas. Second, the SHAPS focuses exclusively on consummatory pleasure and lacks an assessment of anticipatory anhedonia. Third, some confounding clinical variables that may affect the severity of depression symptoms and cognitive style were not investigated. Growing studies have explored the impact of COVID-19 exposures (62), and recent stressful life events combined with cognitive vulnerability can lead to depression (63). In addition, our study did not include information about pharmacological treatments. Considering pharmacological treatment side effects (64, 65) and that anhedonia often persists after antidepressant treatment (66), evaluating drug efficacy and psychosocial factors in future studies will suggest psychotherapy. Fourth, a larger sample size is needed to validate the results.

## Conclusion

Childhood maltreatment, especially emotional neglect, was related to the anhedonia in which sensory experience and social interaction are affected. The dysfunctional attitudes play a mediating role between childhood neglect and anhedonia. For young depression patients with childhood trauma, especially female patients, early cognitive therapy may help to improve the symptoms of anhedonia. Future research needs to explore the impact of childhood traumas on anhedonia with different reward mechanisms and the impact of recent stressful life events.

## Data Availability Statement

The raw data supporting the conclusions of this article will be made available by the authors, without undue reservation.

## Ethics Statement

The studies involving human participants were reviewed and approved by the Ethics Committee of Renmin Hospital of Wuhan University. The patients/participants provided their written informed consent to participate in this study.

## Author Contributions

PW, NZ, LK, and ZL drafted the manuscript. PW, SM, and WW contributed to data analysis, results, and finalized the manuscript. All authors make important contributions to data collection, read, and approved the final manuscript.

## Funding

This work was supported by grants from the National Key R&D Program of China (Grant Number: 2018YFC1314600).

## Conflict of Interest

The authors declare that the research was conducted in the absence of any commercial or financial relationships that could be construed as a potential conflict of interest.

## Publisher's Note

All claims expressed in this article are solely those of the authors and do not necessarily represent those of their affiliated organizations, or those of the publisher, the editors and the reviewers. Any product that may be evaluated in this article, or claim that may be made by its manufacturer, is not guaranteed or endorsed by the publisher.
