# Supplementary material for: Dysfunctional Attitudes Mediate the Relationship Between Childhood Emotional Neglect and Anhedonia in Young Adult Major Depression Patients
Source: Front Psychiatry. 2022 Jan 27;13:791230. doi: 10.3389/fpsyt.2022.791230 (PMC8829348; doi:10.3389/fpsyt.2022.791230)
Supplement: Supplementary file 1 [file Data_Sheet_1.PDF]

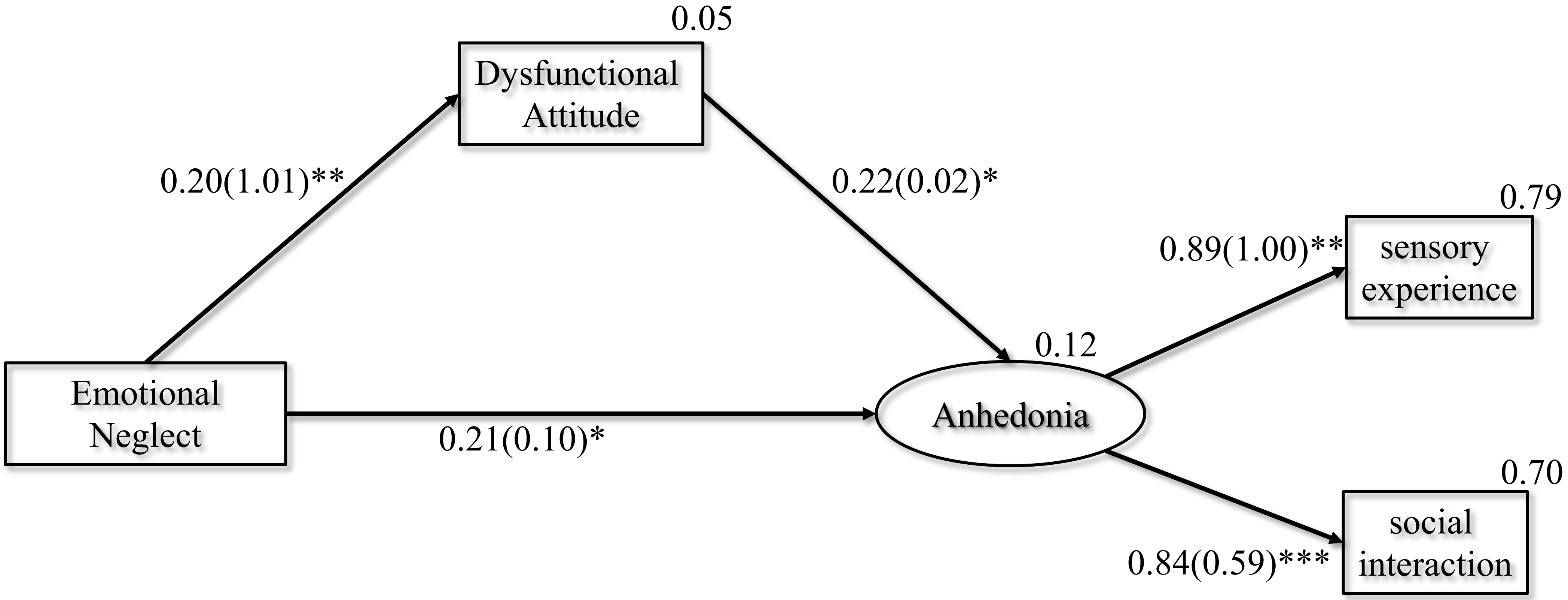

Supplementary Figure 1. Fina path model for female with the standardized and unstandardized coefficients presented in the parentheses.

$\chi^2/df = 1.463$, GFI = 0.993, CFI = 0.994, RMSEA = 0.044, SRMR = 0.0216

$*p < 0.05$, $**\ p < 0.01$, $***\ p < 0.001$

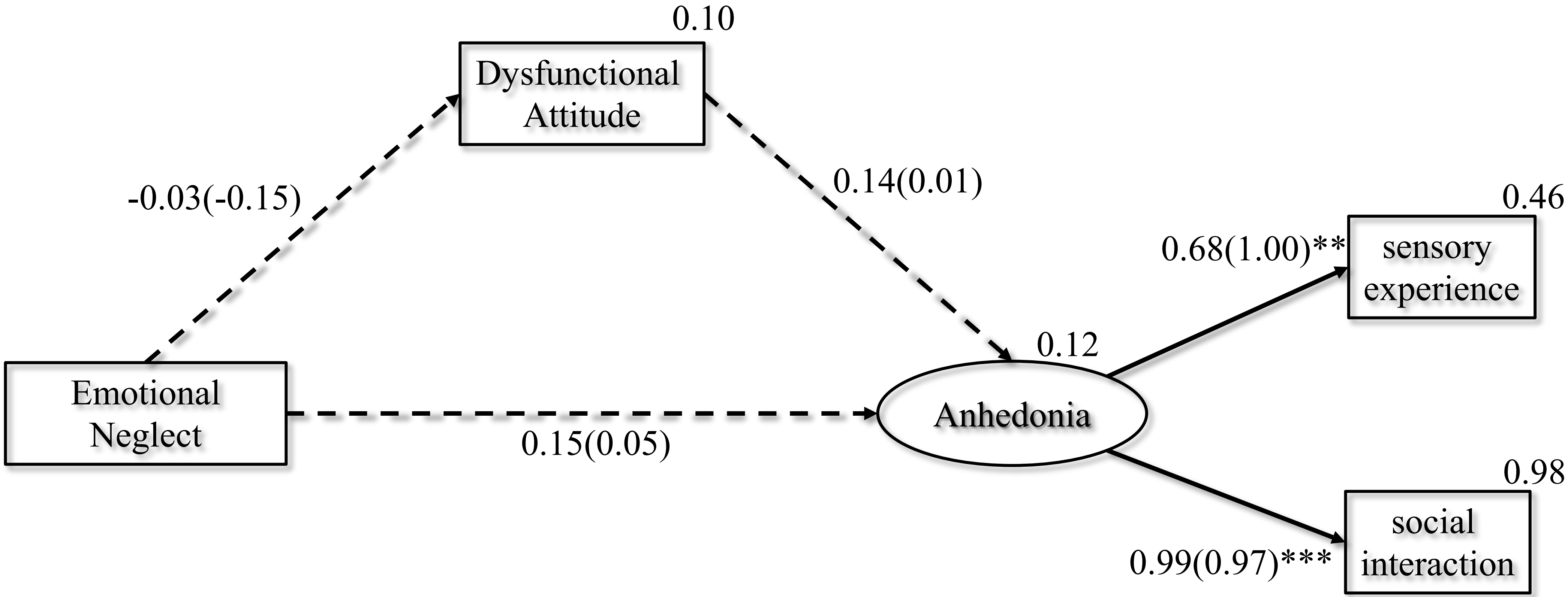

Supplementary Figure 2. Fina path model for male with the standardized and unstandardized coefficients presented in the parentheses.

χ^2/df = 0.918, GFI = 0.983, CFI = 1.000, RMSEA = 0.000, SRMR = 0.0378

The dashed line indicates that the path is not significant.

*$p < 0.05$, ** $p < 0.01$, *** $p < 0.001$

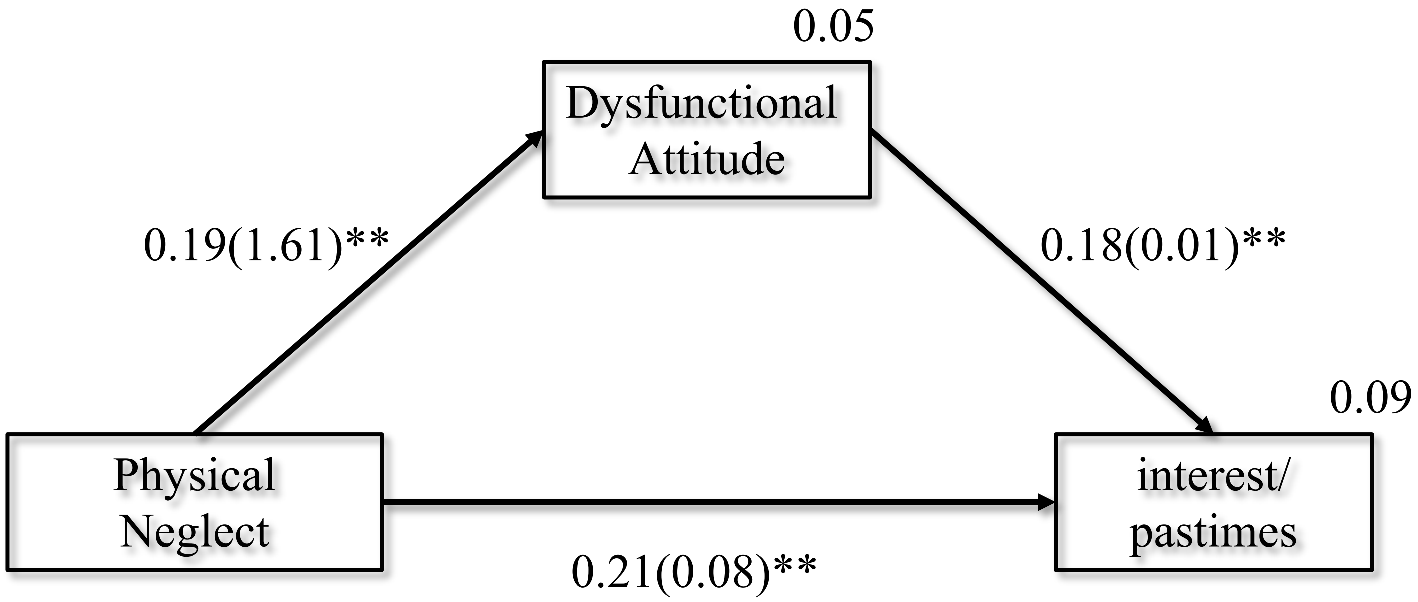

Supplementary Figure 3. Fina path model for female with the standardized and unstandardized coefficients presented in the parentheses.

$\chi^2/df = 0.809$, GFI = 0.998, CFI = 1.000, SRMR = 0.0191

*$p < 0.05$, ** $p < 0.01$, *** $p < 0.001$

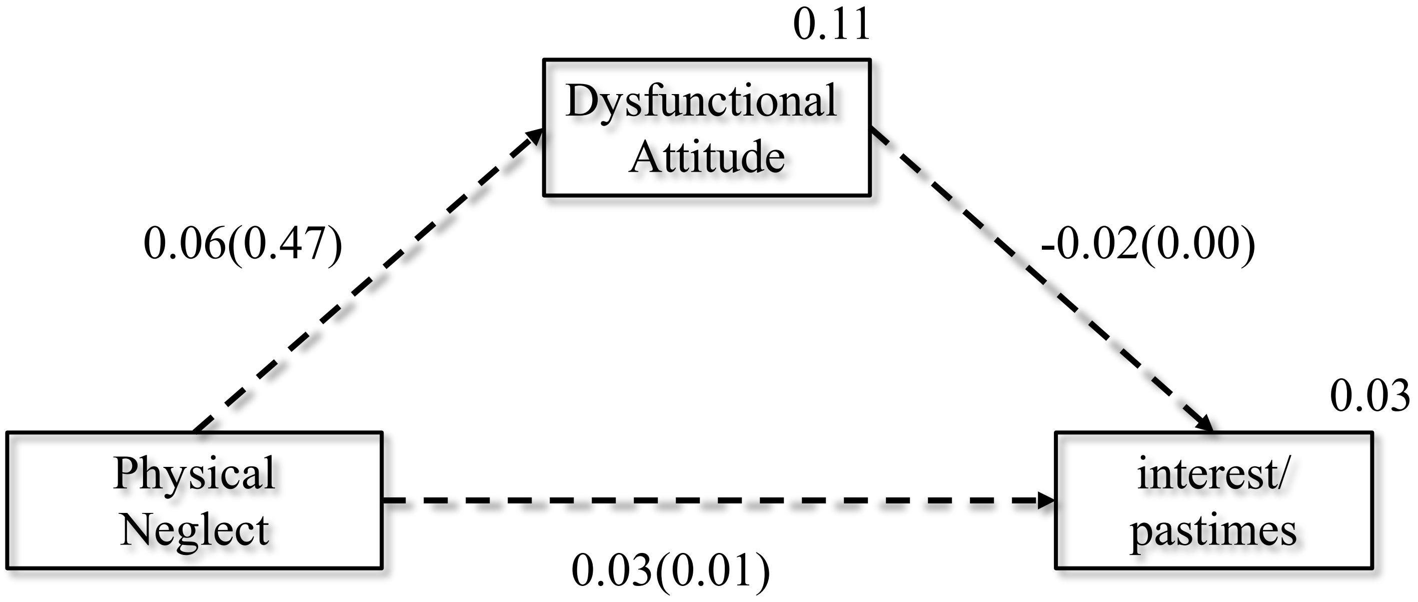

Supplementary Figure 4. Fina path model for male with the standardized and unstandardized coefficients presented in the parentheses.

χ^2/df = 0.068, GFI = 0.999, CFI = 1.000, SRMR = 0.0110

The dashed line indicates that the path is not significant.
